# Enhanced Catalytic Activity of CuO@CuS Core–Shell Structure for Highly Efficient HER Application

**DOI:** 10.3390/nano14231941

**Published:** 2024-12-03

**Authors:** Abu Talha Aqueel Ahmed, Sangeun Cho, Hyunsik Im, Atanu Jana

**Affiliations:** Division of System Semiconductor, Dongguk University, Seoul 04620, Republic of Korea; abutalha.aa@dongguk.edu (A.T.A.A.); sangeun.c@dongguk.edu (S.C.); hyunsik7@dongguk.edu (H.I.)

**Keywords:** hydrothermal growth, CuS, core–shell, HER, nitrogen doping, *TOF*, electrocatalyst

## Abstract

Using electrocatalytic water reduction to produce hydrogen fuel offers significant potential for clean energy, yet its large-scale adoption depends on developing cost-effective, non-precious, and efficient catalysts to replace expensive Pt-based state-of-the-art HER catalysts. The catalytic HER performance of an active catalyst largely depends on the available catalytic active sites, conductivity, and intrinsic electrochemical kinetics, all of which can be altered by incorporating a heteroatom into the active catalyst structure. Herein, we synthesized a unique nitrogen-doped CuO@CuS (NCOS) core–shell-structured catalyst through a facile hydrothermal process followed by an efficacious nitrogenation process, and its electrochemical performance for the HER was systematically analyzed. The NCOS core–shell-structured catalyst exhibits a reduced overpotential (55 mV) and Tafel slope (107 mV dec^−1^) compared to the pure CuS (CS; 179 mV and 201 mV dec^−1^) catalyst at a current density of 10 mA cm^−2^. Moreover, the NCOS core–shell-structured catalyst demonstrates excellent endurance for up to 50 h of chronopotentiometric testing at a driving current density rate of 10 and 100 mA cm^−2^. This excellent catalytic HER activity is a result of an increased electron transfer rate and a greater number of accessible active sites, attributed to a change in structural properties and the high electronic conductivity aroused from nitrogen incorporation, as evidenced from the *TOF* and EIS curve analyses.

## 1. Introduction

The extensive use of fossil fuels creates numerous environmental challenges, most notably contributing to climate modification through greenhouse gas discharge, which contributes to global warming, rising sea levels, extreme weather, the rise in CO_2_ in the atmosphere, and air pollution [1,2,3,4]. To combat these issues, our energy sources must be transformed into an alternative clean and renewable energy eco-system [5,6,7,8]. Among the various renewable sources available, hydrogen (H_2_), with a high energy density of 142 MJ kg^−1^ and zero carbon footprint, is regarded as a promising green fuel that could address global environmental challenges stemming from the excessive utilization of fossil fuels and resource depletion [9,10,11,12,13]. Unlike the traditional steam methane reforming technique, the production of H_2_ fuel from Earth-abundant water through electrolysis (2H_2_O → 2H_2_ + O_2_) is an eminently clean, durable, and efficient method [14,15,16]. Although Pt-based materials are considered the leading electrocatalysts for the HER, their high costs and limited stability hinder their scalability for widespread applications in alkaline electrolyte media [17,18,19,20]. Extensive efforts have been focused on creating Earth-abundant, stable, and inexpensive catalysts to mimic Pt-like HER activity and further the development of water electrolysis as a feasible energy conversion technology, which includes transition-metal phosphides, carbides, sulfides, borides, oxides, and nitrides [21,22,23,24,25,26,27].

Recent developments portray that there has been significant interest in first-row transition-metal sulfides (MSs) for catalytic HER applications. Specifically, Cu-based metal sulfide catalysts, owing to their favorable electronic structure, excellent redox properties, Earth abundancy, and cost-effectiveness, are seen as promising and viable alternatives to state-of-the-art Pt-based catalysts [28,29]. So far, although various Cu-based HER catalysts, such as monometal (e.g., CuO and CuS, etc.), bimetal (e.g., CuCo_2_O_4_ and CuCo_2_S_4_, etc.), core–shell (e.g., CuO@NiP and CuCo_2_O_4_@CQDs, etc.), and heterostructured catalysts (e.g., CuS/NiFe-LDH and Mo-Cu_2_S/CF, etc.), have been extensively explored, their catalytic activity and durability in alkaline solutions remain significantly lower than those of Pt catalysts (Table S1) [14,24,30,31,32,33,34,35,36,37]. However, Cu metal has limited catalytic HER activity because of its inability to effectively capture H atoms, and the unavoidable surface oxidation of MSs in alkaline media deteriorates the overall catalytic performance [28,38]. These challenges can be addressed by modifying the active catalyst’s electronic structure through doping with heterogeneous nitrogen atoms, and creating a core–shell structure could prevent material degradation during long-term testing in alkaline media [39,40,41,42,43,44,45,46,47,48]. Nitrogen bonding with Cu contributes to enhancing the material conductivity because of the higher electronegativity of nitrogen compared to sulfur, and this leads to larger catalytically active sites. Further, nitrogen dopants can reduce the electron-donor capability, modify the surface’s chemical and electronic properties, and result in enhanced electron transportation, leading to boosted electrochemical activity [48,49].

Inspired by the above underlying viewpoints, here, we report on a nitrogen (N)-doped CuO@CuS (NCOS) core–shell-structured catalyst synthesized on a porous three-dimensional (3D) nickel foam (NF) substrate. The design strategy is quite simple yet effective for improving catalytic HER activity in a 1.0 M KOH medium. The XRD, Raman spectroscopy, EDAX, and XPS results confirm the phase-pure formation of CuS (CS), which acts as a template to form the NCOS core–shell-structured catalyst. The detailed synthesis procedure is thoroughly discussed later in the experimental section. The NCOS core–shell-structured catalyst demonstrates outstanding HER performance by achieving low overpotentials of 55 and 179 mV at current densities of 10 and 100 mA cm^−2^, along with a smaller Tafel slope of 107 mV dec^−1^ compared to the pure CS catalyst (121 and 261 mV at 10 and 100 mA cm^−2^, respectively, and 201 mV dec^−1^). The improved HER performance is likely due to the addition of nitrogen and the core–shell structure formed on the NF substrate, which significantly boost catalytic efficiency. The excellent HER activity of the NCOS core–shell-structured catalyst is fairly comparable with the previously reported Cu-based HER catalyst in alkaline 1.0 KOH conditions, which is summarized in Table S1. Moreover, the NCOS core–shell-structured catalyst also demonstrates impressive durability without any significant voltage loss over a prolonged stability (50 h at 10 and 100 mA cm^−2^) test.

## 2. Materials and Synthesis Procedures

### 2.1. Chemical Materials

All chemicals used in the present experiment for the synthesis of the desired catalyst electrode were of analytical grade and were utilized directly without any additional refinement. The chemical reagents, including hydrochloric acid (HCl; 37%), urea (NH_2_CONH_2_; ≥99%), ethanol (CH_3_CH_2_OH; ≥99.50%), potassium hydroxide (KOH; ≥85%, pellets), copper nitrate trihydrate (Cu(NO_3_)_2_·3H_2_O; 99.999%), thiourea (NH_2_CSNH_2_; ≥99.0%), and acetone (CH_3_COCH_3_; ≥99.5%), were purchased from Sigma Aldrich (MERCK, St. Louis, MO, USA). The macroporous 3D NF substrate of thickness 1.6 mm was provided by Alantum (Seoul, Republic of Korea; sheet size of 200 mm × 300 mm).

### 2.2. Synthesis of CS and NCOS

**Figure 1:**
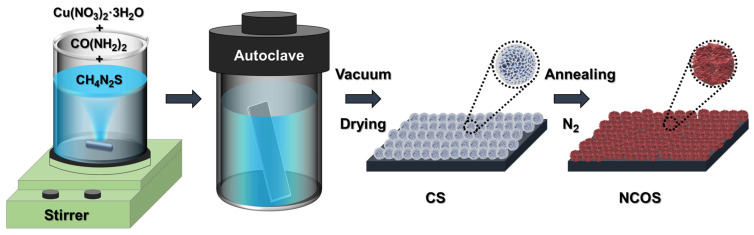
Schematic diagram representing the systematic synthesis process for the production of CuS (CS)- and nitrogen (N)-doped CuO@CuS (NCOS) core–shell-structured catalysts.

The fabrication procedure for the CS and NCOS core–shell catalysts is similar to that used in our previous report, which is schematically illustrated in Figure 1, portraying the CS and NCOS catalysts with distinct surface textures [50]. In our synthesis procedure, the CS was initially grown on the pre-cleaned macroporous 3D surface of an NF substrate (electrode size of 10 mm × 50 mm with exposed area of 10 mm × 10 mm) using a hydrothermal procedure. The formed CS electrode film was then treated under a nitrogen (N_2_) atmosphere to form the NCOS core–shell structure. The detailed process is as follows: First, 5 mmol of Cu(NO_3_)_2_·3H_2_O and 30 mmol of NH_2_CONH_2_ were dissolved in a glass beaker containing 50 mL of DI water under vigorous stirring. Thereafter, 50 mmol of NH_2_CSNH_2_ was then added to this mixture until the solution became transparent at ambient room temperature (R. T.). The pre-cleaned NF substrate and the transparent mixture solution were transferred into a 0.1 L Teflon vessel in a stainless-steel autoclave. The autoclave was then sealed and moved to a muffle furnace, and the reaction was executed at a constant temperature of 160 °C for about 12 h. After undisturbed cooling up to R. T., the obtained electrode film was collected and washed with DI water and CH_3_CH_2_OH quite a few times, followed by overnight drying at 80 °C in a vacuum at ambient pressure. Subsequently, the formed CS electrode film was treated under a sealed N_2_ ambient atmosphere in a tubular furnace at 350 °C for 2 h to obtain the desired NCOS core–shell-structured electrode film.

### 2.3. Characterization of the Material

The formed electrode material’s phase, crystallinity, and structural characteristics were examined by an X-ray diffraction (XRD) spectral analysis technique. The Rigaku Smartlab (Tokyo, Japan) instrument was used to obtain the XRD spectra of both electrode films using the radiation of a CuKα source with a proceeding wavelength of 1.54056 Å. The XRD spectra were measured with a 2° min^−1^ scanning rate in a spectral angle (2θ) range between 20° and 80°. The material impressions and the constituent elemental bonds were scrutinized through Raman spectroscopy. The LabRam Armis instrument (Jobin Yvon, Longjumeau, France) was implemented to obtain the Raman spectra of the films by utilizing an Ar-ion laser beam with a wavelength of 514 nm. The Raman spectra were recorded in a frequency range between 200 cm^−1^ and 675 cm^−1^. The material morphology at low and high magnifications was recorded using field emission scanning electron microscopy (FE-SEM), and the chemical composition of the constituent elements was examined with the help of energy-dispersive X-ray spectroscopy (EDAX). The JEOL JSM-6701F instrument (Tokyo, Japan) was used to record the FE-SEM images, EDAX spectra, and EDAX mapping (×5000 magnification). The constituent elements’ oxidation states were evaluated through X-ray photoelectron spectroscopy (XPS). Further, the XPS spectra were obtained with a ULVAC PHI 5000 VersaProbe instrument (Kanagawa, Japan). The elemental binding energies (B. E.) of the XPS spectra were rectified using the contaminant carbon (C 1s at 284.23 eV) emission signal existing inside the vacuum chamber of the instrument.

### 2.4. Electrochemical HER Evaluation

The electrocatalytic HER activities of the prepared CS and NCOS electrode films were evaluated using various electrochemical measurements such as linear sweep voltammetry (LSV), chronopotentiometry, non-Faradaic cyclic voltammetry (CV), and electrochemical impedance–spectroscopy (EIS) curves. A standard three-electrode VersaSTAT instrument (Ametek Scientific Instruments, Berwyn, PA, USA) was employed to examine the characteristic catalytic HER performances in a cell containing a 1.0 M KOH electrolyte medium. The electrochemical cell was fabricated using the synthesized electrode (CS and NCOS) films, Pt foil, and a saturated calomel electrode (SCE) filled with KCl as the working, counter, and reference electrodes, respectively. The LSV curves were measured at a scan rate (*v*) of 1.0 mV s^−1^ in a voltage range between 0.0 and −1.5 V (vs. SCE). To obtain the HER overpotential, the reference SCE scale of the obtained potential was changed into a reversible hydrogen electrode (RHE) scale, and, simultaneously, ohmic loss was then compensated for using the electrolyte and internal resistance (*R*s) within the electrochemical system as follows:

*E*_RHE_ = (pH × 0.059) + *E*°_SCE_ + *E*_SCE_, (1)

*E*_RHE_ (*JR*s_-corrected_) = *E*_RHE_ − (*R*s × *J*), (2)

where *E*_RHE_ is the obtained voltage in the RHE reference scale, pH is the potential of hydrogen, *E*°_SCE_ is the standard potential of SCE at R. T., *E*_SCE_ is the measured voltage on the SCE scale, and *J* is the driven current density. The overpotential (*ɳ*) was then estimated from the *JR*s_-corrected_ LSV curves using the following equations:

*ɳ* = *E*_RHE_ (*JR*s_-corrected_), (3)

*ɳ* = [*β* × log (*J*)] + α, (4)

where *β* is the Tafel slope obtained from the linear section of the LSV curve, and α is the constant of Equation (4). Moreover, the charge transfer characteristics during the electrochemical HER test were examined using electrochemical impedance spectroscopy (EIS). The EIS curves were recorded over a wide frequency range between 0.01 and 10 kHz with an applied AC signal amplitude of 10 mV and a negative biasing potential of 400 mV.

## 3. Results and Discussion

### 3.1. Crystallographic and Compositional Properties

**Figure 2:**
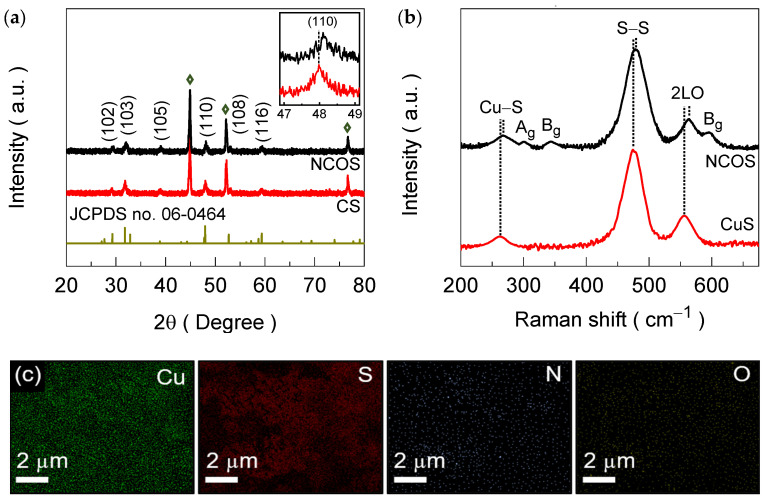
(**a**) XRD spectra of the CS and NCOS core–shell-structured electrode films measured in a spectral angle range between 20° and 80°. Notably, the additional three intense XRD peaks in the spectra arise from the NF substrate, indicated by diamond symbols. (**b**) Raman spectra for the CS and NCOS core–shell-structured electrode films. (**c**) EDAX image mapping of the constituent S, Cu, N, and O elements for the NCOS core–shell-structured electrode films.

The structural characteristics of the bare CS and NCOS electrode films were investigated through XRD measurements to understand the effect of the N-dopant on the material structure. Figure 2a depicts the XRD spectra of both the pure CuS and NCOS electrode films synthesized on the 3D NF substrate accompanied by a reference JCPDS card pattern (JCPDS card no. 06-0464). The extra three intense XRD peaks depicted as diamond symbols in the XRD spectra originate from the NF substrate. Characteristic diffraction peaks for the CS electrode film were observed at 29.12, 31.83, 38.94, 47.98, 53.11, and 59.35°, ascribed to the (102), (103), (105), (110), (108), and (116) crystal planes of CuS, respectively [51]. After nitrogen treatment, the NCOS electrode film revealed XRD peaks, which were very similar to those of the CS electrode film. However, a notable positive rigid shift of ~ 0.12° was observed towards higher values, and the respective diffraction peaks were slightly broadened, as evidenced from the increase in the full-width at half-maximum (FWHM). The observed shift in the diffraction peaks and the simultaneously widened FWHM might have occurred due to a reduction in the interplanar spacing owing to the smaller atomic ratio of the N-dopant, suggesting the efficacious incorporation of N atoms within the crystal lattice [52].

Thereafter, the vibrational characteristics of the CS and NCOS electrode films were examined through Raman measurements. Figure 2b shows the obtained Raman spectra, which reveal three common vibrational peaks located at 262 (Cu–S vibration), 475 (S–S ions symmetrically stretching at 4e site), and 556 (longitudinal overtone of phonons) cm^−1^, confirming the formation of CuS [53]. However, these identical vibrations for the NCOS electrode film were moderately blue-shifted compared to those of the CS electrode film because of the increased vibrational frequency resulting from the altered surface state during nitrogen incorporation. Apart from these characteristic Raman vibrations, the NCOS Raman spectrum reveal three additional noticeable peaks. The first Raman peak at 301 cm^−1^ corresponds to the A_g_ vibrational mode, and the latter two peaks at 343 and 595 cm^−1^ are associated with the B_g_ vibrational mode of amorphous CuO [54,55]. The presence of CuO, along with the increased CuS phase, could possibly have arisen due to surface oxidation upon high-temperature annealing. To validate the above results, EDAX analysis was then conducted for both the CS and NCOS electrode films. Figure S1 shows the EDAX spectra, which exhibit the stoichiometric ratios of the Cu and S constituents in the CS spectrum and traces of N and O along with the constituent Cu and S in the NCOS spectrum. Further, EDAX image mapping for the NCOS electrode film was also performed. Figure 2c shows the obtained EDAX mapping image for the NCOS electrode films, exhibiting the uniform presence of N (2.36%), S (45.17%), Cu (48.82%), and O (3.65%).

### 3.2. Chemical State Characteristics

**Figure 3:**
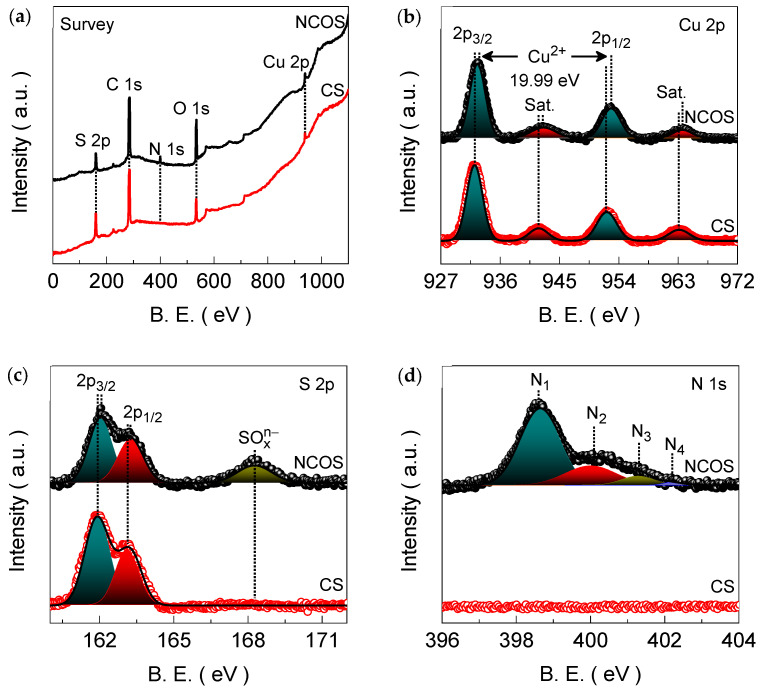
A comparative (**a**) survey and the narrow-ranged (**b**) Cu 2p, (**c**) S 2p, and (**d**) N 1s XPS spectra for the CS and NCOS core–shell-structured electrode films. The narrow-ranged emission spectra were deconvoluted by a Gaussian peak fitting model.

The XPS spectra for the CS and NCOS electrode films were then recorded to analyze the elemental binding states. Figure 3a shows the full-range survey spectra of the CS and NCOS electrode films, exhibiting characteristic Cu, S, O, and N emissions. The high-resolution emission spectra for these elements were then recorded to evaluate the binding states of the constituent elements. Figure 3b displays the Cu 2p emission spectra of the CS and NCOS electrode films. Four typical emission peaks deconvoluted at 932.21 (Cu 2p_3/2_), 941.87 (satellite; Sat.), 952.17 (Cu 2p_1/2_), and 963.14 (Sat.) eV were observed for the CS electrode film. The separation of spin–orbit splitting at 19.96 eV (~20 eV) for the degenerate Cu 2p_3/2_ and Cu 2p_1/2_ states and the characteristic satellite emission peak confirm the presence of Cu^2+^ in the CuS structure [24,56]. For the CS electrode film, the high-resolution S 2p emission (Figure 3c) spectrum displays two deconvoluted peaks at 161.92 (S 2p_3/2_) and 163.11 (S 2p_1/2_) eV, with the 1.19 eV spin-energy separation of the characteristic peaks confirming the existence of the S^2−^ state in the CuS structure [56,57]. Although the NCOS electrode film reveals a similar spin-energy separation of degenerate states, the peak positions of the distinctive 2p_3/2_ and 2p_1/2_ degenerate states of the characteristic Cu 2p and S 2p emissions were moderately blue-shifted compared to those for the CS electrode film. This change in the binding energy position is a result of the successful attachment of N atoms to the CuS structure because the incorporated N atoms reduce the electron density around the Cu atom and introduce lattice distortions, resulting in the peak shifting toward higher binding energies [58]. Moreover, the S 2p spectrum of the NCOS electrode film reveals an additional broad peak at 168.28 eV (SO_x_^n−^) as a result of surface oxidation, which is in good agreement with the increased O 1s peak intensity for the NCOS electrode compared to the CS electrode (Figure S2) [38]. Further, the NCOS electrode film only shows N 1s emission (Figure 3d), which is deconvoluted into four peaks at 398.63 (pyridinic N; metal nitrogen bonding), 400.03 (pyrrolic N), 401.28 (graphitic N), and 402.22 (oxidized N) eV [58,59,60,61].

### 3.3. Morphological Properties

**Figure 4:**
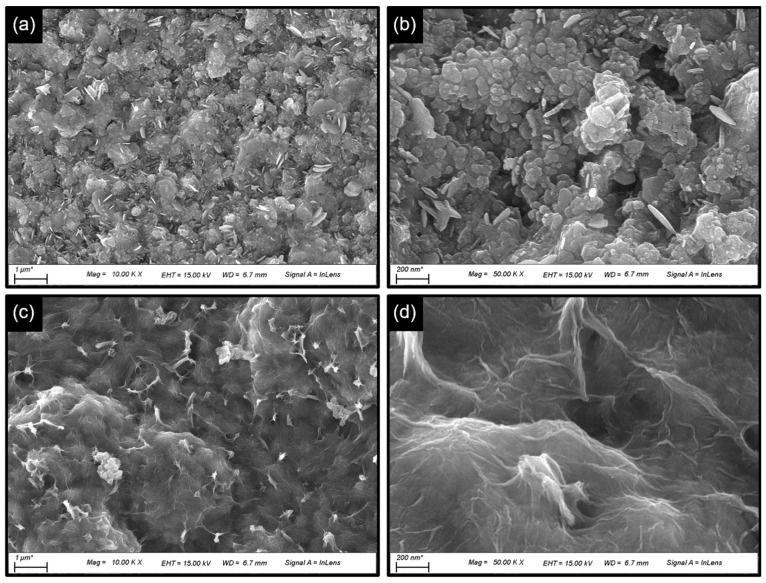
FE-SEM images of (**a**,**c**) CS and (**b**,**d**) NCOS core–shell-structured electrode films synthesized on the NF substrate. The FE-SEM images were captured at low and high magnifications.

The microstructure features and surface topography of the electrode films were examined by FE-SEM measurements. Figure 4a,b show the FE-SEM images of the CS electrode film, which display a densely packed spherical morphology with a randomly embossed textured surface. The average size of these spherical particles ranges between approximately 300 and 400 nm (Figure 4b). Figure 4c shows the FE-SEM images of the NCOS electrode film, which retains a similar morphology as that of the CS electrode film. However, the surface texture undergoes significant changes following nitrogen incorporation. A distinct flake-like texture (i.e., CuO shell, Figure 4d) covers the entire surface of the CS spheres (i.e., core), which might appear due to recrystallization upon surface oxidation during high-temperature heating. This ultrathin nanoflake-textured shell envelops all the interconnected CuS spheres, creating a core–shell structure, which could help to increase the catalytically active region and preserve the catalytic HER activity of the core CuS catalyst in alkaline KOH conditions.

### 3.4. Electrochemical HER Performance

**Figure 5:**
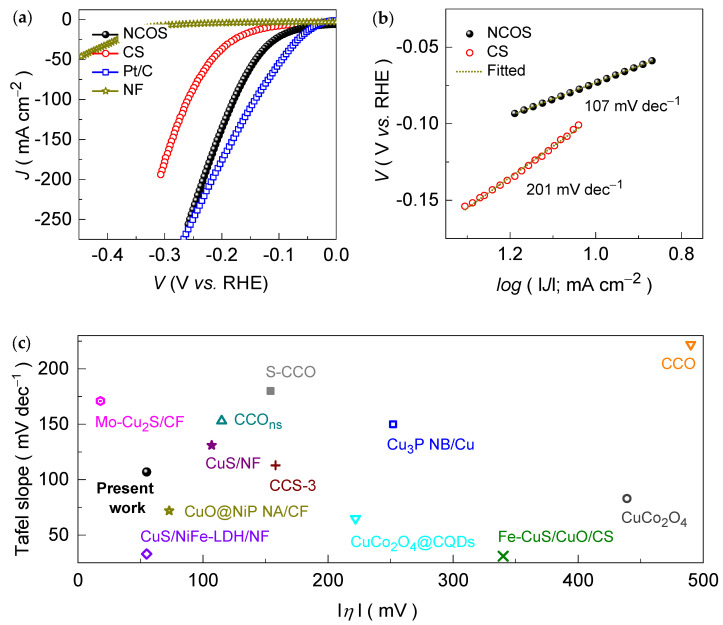
(**a**) LSV curves for the CS, NF, and NCOS core–shell-structured catalysts measured at 1.0 mV s^−1^ in alkaline 1.0 KOH medium. (**b**) Tafel plots for the CS and NCOS core–shell-structured catalysts. (**c**) A comparison of the HER performance of the NCOS core–shell-structured catalyst with that of the previously reported Cu-based metal-oxide/sulfide catalysts at 10 mA cm^−2^, evaluated in an alkaline 1.0 M KOH electrolyte medium.

Figure 5a shows the catalytic HER activity of the CS and NCOS core–shell-structured catalysts grown on the porous 3D architecture of NF. The NCOS core–shell-structured catalyst synthesized on the NF reveals an overpotential of 55 mV at a current density of 10 mA cm^−2^. In contrast, the pure CS catalyst on NF exhibits a larger overpotential of 121 mV at the same driving current density. Further, at a current density of 100 mA cm^–2^, the NCOS core–shell-structured catalyst achieves an overpotential of 179 mV and exhibits a Faradaic efficiency of ~98%, which are superior compared to the overpotential and Faradaic efficiency of the pure CS catalyst (261 mV at 100 mA cm^−2^ and a Faradaic efficiency of 87%). These findings indicate that N-doping can substantially enhance the HER activity of an active catalyst material. The HER activity of the commercial Pt/C and pure NF substrates was also measured for comparison in a 1.0 M alkaline KOH electrolyte medium at 1.0 mV s^−1^. The overpotential of the noble metal Pt/C catalyst is closely comparable to that of the synthesized NCOS core–shell-structured catalyst at a higher driving current density. However, an infinitesimally small voltage response was noted for the NF substrate over the entire potential scanning range compared to the synthesized catalysts, suggesting that the NF substrate itself does not play a major role in enhancing catalytic activity. Instead, it provides a large surface area for catalyst loading, which enables a more accessible surface of the deposited catalysts for the electrochemical process, and possesses sufficient mechanical stability to withstand prolonged testing. In addition, to further confirm the above results, a series of catalyst electrodes were prepared and examined in the KOH medium (Figure S3). These catalysts demonstrated consistent HER activity across multiple tests under identical conditions, confirming the reliability of the obtained catalytic HER performance.

Generally, the electrochemical HER process starts with proton discharge on the metal surface, where the water dissociates to form adsorbed hydrogen (MH_ads_) and hydroxide ions (OH^−^), which is known as the Volmer reaction (M + H_2_O + e^−^ → MH_ads_ + OH^−^, Tafel slope = 120 mV dec^−1^) [62]. This reaction step is either followed by electrochemical desorption, where MH_ads_ reacts with water to release H_2_, which is known as the Heyrovsky reaction (MH_ads_ + H_2_O + e^−^ → M + OH^−^ + H_2_, Tafel slope = 40 mV dec^−1^), or by chemical desorption, where two MH_ads_ species recombine to release H_2_, which is known as the Tafel reaction (2MH_ads_ → 2M + H_2_, Tafel slope = 30 mV dec^−1^). Therefore, to understand the catalytic superiority of the NCOS core–shell catalyst compared to the CS catalyst, Tafel curves (Figure 5b) were procured from the obtained LSV curves (Figure 5a). The NCOS core–shell-structured catalyst exhibits a reduced Tafel slope of 107 mV dec^−1^ compared to the pure CS (107 mV dec^−1^) catalyst. The decrement in the overpotential is associated with the enhanced reaction kinetics of the active catalyst after nitrogen treatment. The incorporated nitrogen atoms alter the electronic structure of the catalyst, which results in improved electronic conductivity and further helps to increase the electrochemically active surface area (*ECSA*, Figure S4a–c), thereby enhancing the rate of the reaction kinetics. The excellent electrocatalytic HER performance of the NCOS core–shell-structured catalyst in the KOH electrolyte medium is fairly comparable to that of the previously reported Cu-based metal-oxide/sulfide catalysts (Figure 5c and Table S1).

**Figure 6:**
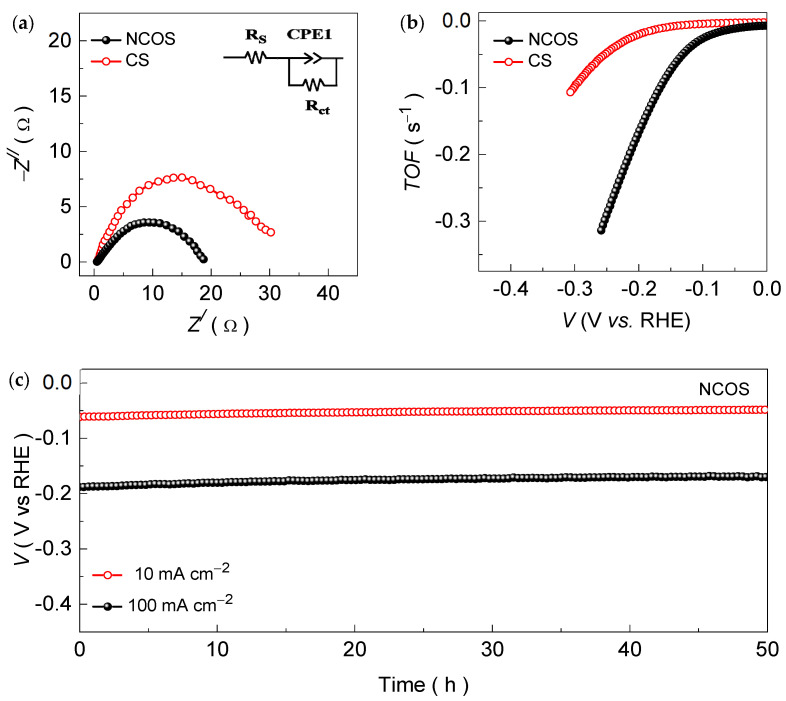
(**a**) EIS and (**b**) *TOF* curves for the CS and NCOS core–shell-structured catalysts. (**c**) Long-term chronopotentiometric HER stability curves for the NCOS core–shell-structured catalyst measured for up to 50 h at current densities of 10 and 100 mA cm^−2^.

To gain insight into the altered electronic conductivity and electron transfer kinetics after nitrogen incorporation, EIS (Nyquist impedance) curves were measured for both catalysts. Figure 6a shows the EIS plots for the CS and NCOS core–shell-structured catalysts. The obtained EIS curves exhibit a semicircular curvature and were fitted through the tank circuit using Z-view software. The inset of Figure 6a shows the modeled tank circuit and the obtained parameter values, which are summarized in Table 1. The intersection of the semicircular curvature with the Zʹ-axis (i.e., X-axis) represents *R*s, and the semicircular curvature is a measure of the charge transfer resistance (*R*_ct_) [38]. Therefore, the value of *R*_ct_ highlights how easy electron transfer is at the electrode/electrolyte interface. The NCOS core–shell-structured catalyst demonstrates an *R*s and *R*_ct_ of 0.503 and 18.17 Ω, respectively. Meanwhile, the pure CS catalyst exhibits comparatively higher *R*s (0.612 Ω) and *R*_ct_ (29.34 Ω) values than the NCOS catalyst. This is because the N-dopant allows for a higher number of accessible catalytically active sites (Figure S4) and contributes to lowering the energy barrier for electrochemical HER processes, which results in kinetically efficient electron/ion transportation in the core–shell structure and boosts the electrocatalytic efficiency.

Thereafter, the turnover frequency (*TOF*) of the CS and NCOS core–shell-structured catalysts was estimated to further understand the intrinsic HER kinetics and catalytic efficiency. The *TOF* quantifies the number of molecules reacting at the accessible electrochemically active sites per unit time, and it can be estimated as follows [38]:

*TOF* = [(*A* × *J*)/(2 × *F* × *n*)], (5)

where *A* is the loading area of the active catalyst material (incm^2^), the factor of 2 is due to the two electrons involved per mole of H_2_ in the HER, *F* is the Faraday constant (96,485.3329 A·s mol^−1^), and *n* is the number of moles of the active catalyst. Figure 6b shows the *TOF* curves for the CS and NCOS core–shell-structured catalysts obtained from the measured LSV curves using the above Equation (5). The NCOS core–shell-structured catalyst demonstrates a *TOF* of 0.313 s^−1^, which is significantly higher compared to the CS (0.055 s^−1^) catalyst at the same driving voltage. The increased *TOF* is a result of the improved HER kinetics after nitrogen substitution, which enable more efficient electron and ion transport across the catalyst network and are in good agreement with the Tafel (Figure 5b) and *ECSA*-corrected LSV curves (Figure S4d).

Owing to the overall electrocatalytic superiority of the NCOS catalyst compared to the CS catalyst, the stability of the NCOS core–shell structure was then evaluated to examine the catalysts’ endurance over a wide range of current density rates in an alkaline KOH (1.0 M) medium. Figure 6c shows the long-term chronopotentiometric stability of the NCOS core–shell-structured catalyst measured at applied current densities of 10 and 100 mA cm^−2^. Interestingly, the NCOS core–shell-structured catalyst demonstrated a static potential response during vigorous and continuous H_2_ gas bubble evolution throughout the long-term chronopotentiometric test without any significant deviation in the voltage response at both driven current densities for up to 50 h, confirming its long-term durability for the catalytic HER in an alkaline KOH medium. Its excellent stability endurance is further supported by the post-stability-measured EIS and LSV curves (Figure S5), which were measured under the same experimental conditions. Insignificant variations in the charge transfer resistance and voltage response were observed for the NCOS core–shell-structured catalyst before and after the long-term chronopotentiometric stability test, which was a result of superior electron and ion transport, contributing to the strong chronopotentiometric stability and highlighting the robustness of the core–shell-structured catalyst for catalytic HER performance. The NCOS core–shell-structured catalyst, after the accelerated long-term chronopotentiometric test, was characterized by Raman and EDAX spectrum analyses (Figure S6), which suggest that the prolonged stability evaluation did not notably change the material bindings and compositions of the constituent (Cu, S, N, and O) elements. The excellent stability of the NCOS core–shell-structured catalyst was likely due to the addition of nitrogen, which altered the electronic structure of the formed catalyst, enabling efficient electron/ion transport, and the formed core–shell structure prevented material degradation during chronopotentiometric stability testing in the KOH medium.

## 4. Conclusions

In summary, we incorporated nitrogen into the CS structure through a simple but effective nitrogenation method. The CS structure was prepared on the microporous architecture of 3D NF via a hydrothermal process to enhance the electrochemical HER performance of the active catalyst. The physical, morphological, and chemical characteristics confirmed the phase-pure formation of CS and its successful transformation into the unique NCOS core–shell-structured catalyst. Nitrogen incorporation significantly transformed the surface topography into a flake-like textured shell on the CS surface, which might have helped to protect the core from degradation, enhancing its chronopotentiometric stability during the catalytic HER. The catalytic activity of the NCOS core–shell-structured catalyst was significantly enhanced, demonstrating lowered overpotentials of 55 and 179 mV at 10 and 100 mA cm^–2^, respectively, compared to the CS (121 and 261 mV) catalyst. The boosted catalytic activity of the NCOS catalyst is linked to improved intrinsic reaction kinetics, efficient electron/ion transport, and a higher number of catalytically active sites after nitrogenation, as evidenced from the Tafel, *TOF*, normalized *J_ECSA_*, EIS, and *ECSA* curve analyses. Further, the insignificant change in the voltage response during the long-term chronopotentiometric stability test over 50 h confirms its excellent endurance at low and high current density rates of 10 and 100 mA cm^–2^.

## Figures and Tables

**Table 1:** The EIS curve fitting parameters for the CS and NCOS core–shell-structured catalysts.

Catalyst Electrode	Pre-HER Stability		Post-HER Stability	
	Rs (Ω)	Rct (Ω)	Rs (Ω)	Rct (Ω)
CS	0.612	29.34	-	-
NCOS	0.503	18.17	0.516	18.95

## Data Availability

The data from this study can be accessed upon reasonable request to the corresponding author.

## Supplementary Materials

The supplementary materials for this study are available for download at the following link: https://www.mdpi.com/article/10.3390/nano14231941/s1, Figure S1: EDS spectra; Figure S2: O 1s XPS spectra; Figure S3: Reliability of HER activity at 10 and 100 mA cm^–2^; Figure S4: non-Faradaic CV curves for the estimation of ECSA and ECSA-corrected LSV curves; Figure S5: post-stability-recorded Nyquist impedance and LSV curves; Figure S6: post-stability-recorded EDS and Raman spectra. Table S1. Comparative catalytic HER performance of our optimized NCOS core–shell-structured catalyst and other Cu-based catalyst in alkaline KOH (1.0 M) electrolyte medium at 10 mA cm^–2^. Table S2. Comparative electrochemical double-layer capacitance and ECSA for the CS and NCOS core–shell-structured catalysts.
