# Supplementary material for: Enhanced Catalytic Activity of CuO@CuS Core–Shell Structure for Highly Efficient HER Application
Source: Nanomaterials (Basel). 2024 Dec 3;14(23):1941. doi: 10.3390/nano14231941 (PMC11644029; doi:10.3390/nano14231941)
Supplement: Supplementary file 1 [file nanomaterials-14-01941-s001.zip › nanomaterials-3342797-supplementary.pdf]

Supporting Information

Enhanced Catalytic Activity of CuO@CuS Core–Shell Structure for Highly Efficient HER Application

Abu Talha Aqueel Ahmed[1], Hyunsik Im [1], Sangeun Cho [1], Atanu Jana [1*]

Division of System Semiconductor, Dongguk University, Seoul 04620, Republic of Korea

Corresponding Author: atanujana@dongguk.edu

Table S1. Comparative catalytic HER performance of our optimized NCOS core-shell structured catalyst and other Cu-based catalyst in alkaline KOH (1.0 M) electrolyte medium at 10 mA cm^{-2}.

<table>
<tr><th>No.</th><th colspan="2">Catalyst film</th><th>Overpotential
@100 (mA cm^{-2})</th><th>Tafel slope
(mV dec $^{-1}$)</th><th>Stability at J
(J in mA cm^{-2})</th><th>Supporting Reference</th></tr>
<tr><td>1</td><td colspan="2">CuO@NiP NA/CF</td><td>73 mV</td><td>72</td><td>35 h@0.1 V</td><td>31</td></tr>
<tr><td>2</td><td colspan="2">CCO_{nS}</td><td>115 mV</td><td>153</td><td>30 h@10, 50, and 100</td><td>24</td></tr>
<tr><td>3</td><td colspan="2">CCS-3</td><td>158 mV</td><td>113</td><td>20 h</td><td>32</td></tr>
<tr><td>4</td><td colspan="2">Cu_3P NB/Cu</td><td>252 mV</td><td>150</td><td>10 h@0.42 mV</td><td>33</td></tr>
<tr><td>5</td><td colspan="2">$CuCo_2O_4$</td><td>439 mV</td><td>83</td><td>-</td><td rowspan="2">34</td></tr>
<tr><td>6</td><td colspan="2">$CuCo_2O_4$@CQDs</td><td>222 mV</td><td>65</td><td>1000 cycle@10 mV s^{-1}</td></tr>
<tr><td>7</td><td>CCO</td><td rowspan="2">0.1 M NaOH</td><td>490 mV</td><td>222</td><td rowspan="2">10000 s</td><td rowspan="2">35</td></tr>
<tr><td>8</td><td>S-CCO</td><td>154 mV</td><td>180</td></tr>
<tr><td>9</td><td colspan="2">CuS/NiFe-LDH/NF</td><td>55 mV</td><td>33</td><td>-</td><td rowspan="2">36</td></tr>
<tr><td>10</td><td colspan="2">CuS/NF</td><td>107 mV</td><td>131</td><td>-</td></tr>
<tr><td>11</td><td colspan="2">Fe–CuS/CuO/CS</td><td>340 mV</td><td>31</td><td>30 h@100</td><td>14</td></tr>
<tr><td>12</td><td colspan="2">Mo-Cu_2S/CF</td><td>18 mV</td><td>171</td><td>20 h@60</td><td>37</td></tr>
<tr><td>13</td><td colspan="2">NCOS core-shell structure</td><td>55 mV</td><td>107</td><td>50 h@10
50 h@100</td><td>Present Work</td></tr>
</table>

Supporting Figures

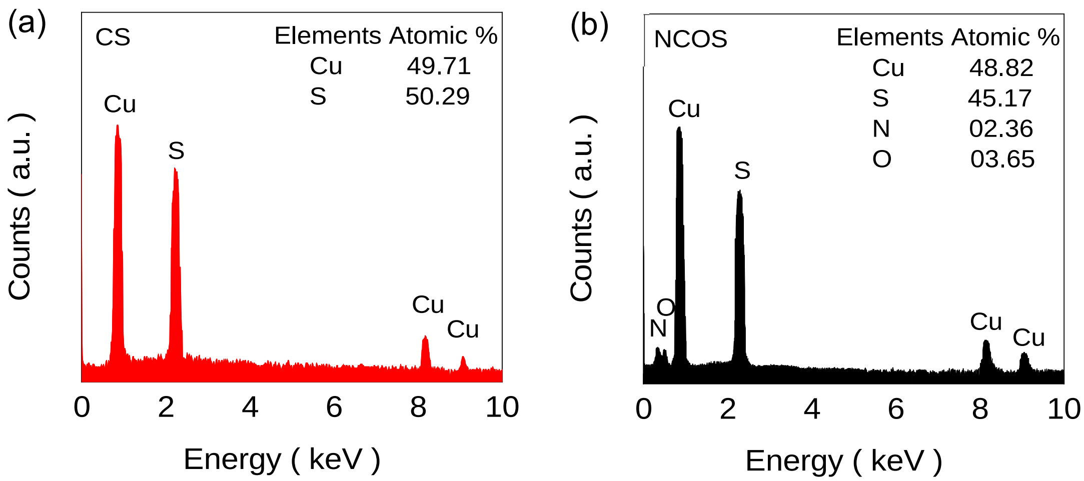

Figure S1. EDS spectra of (**a**) CS and (**b**) NCOS core-shell structure electrode films. The inset table displays the elemental compositions obtained in atomic percentage ratios.

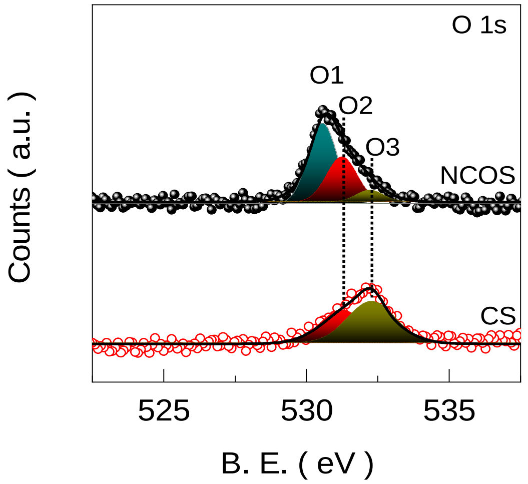

Figure S2. A comparative narrow ranged O 1s XPS spectra for the CS and NCOS core-shell structured electrode films.

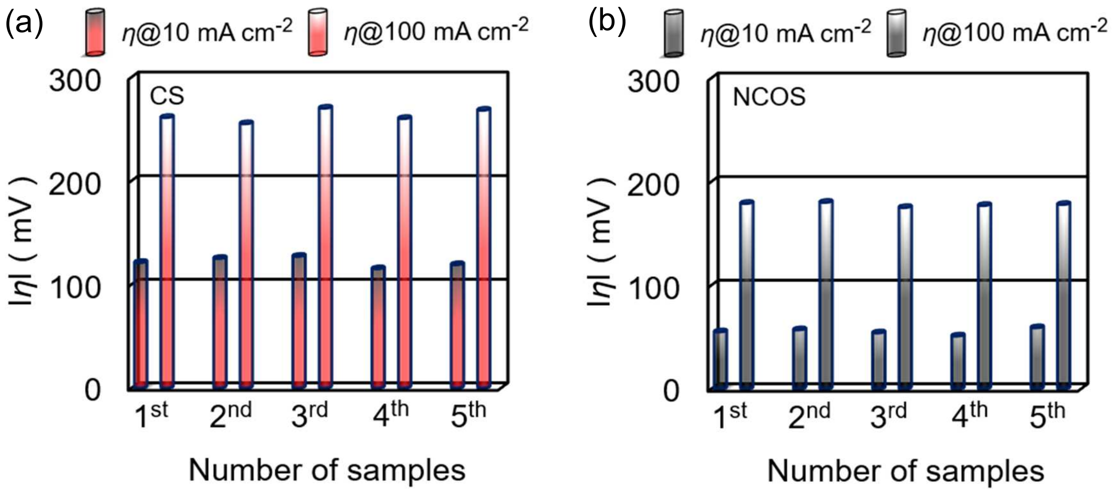

Figure S3. The reliability performance of the (**a**) CS and (**b**) NCOS core-shell structured catalysts.

The electrochemically active surface area (ECSA) represents the available catalytically active sites of a catalyst material. It can be determined by calculating the electrochemical double-layer capacitance at different scan rates within the non-Faradaic region (0.00 to 0.10 V vs. RHE) of the CV curve using the following equations:

$$C_{DL} = J_{dl} / v, \tag{1}$$

$$ECSA = C_{dl} / C_e, \tag{2}$$

where C_{dl}, v, J_{dl}, and C_e (0.04 mF cm^{-2}) are the double-layer capacitance, scan rate, current density in the non-Faradaic region, and the electrolyte solution capacitance, respectively. The C_{dl} and *ECSA* were obtained from the scan rate dependent CV curves using the equations (1) and (2) are summarized in Table S2. The NCOS core-shell structured catalyst exhibits the higher C_{dl} and *ECSA* compared to the CS catalyst, suggesting that the incorporation of nitrogen and the formed flake-like shell facilitates the catalytically active site.

Table S2. Comparative electrochemical double-layer capacitance and ECSA for the CS and NCOS core-shell structured catalysts.

Catalyst electrode	C_{dl} (mF)	*ECSA* (cm^2)
CS	5.84	146
NCOS	9.25	~ 231

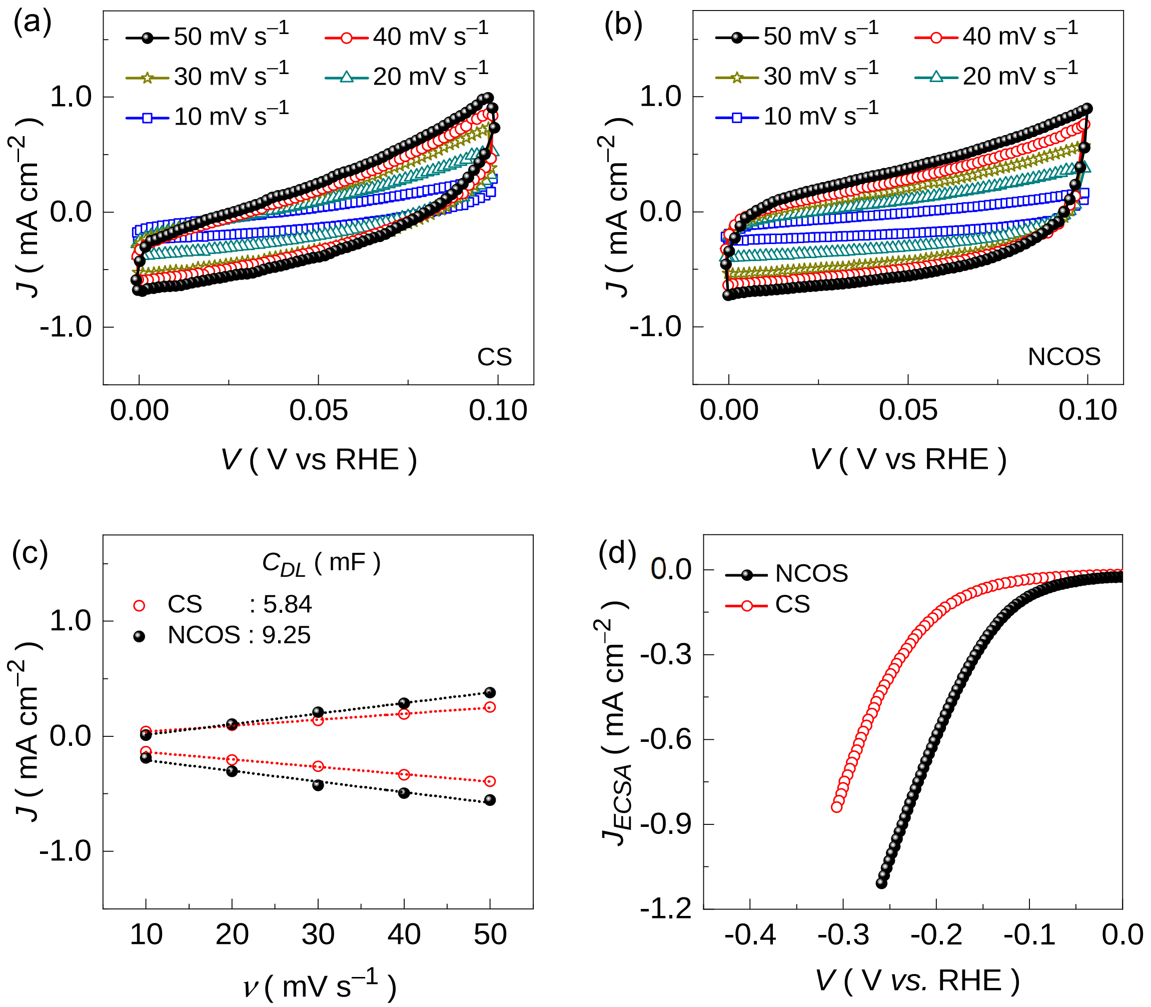

Figure S4. Scan rate dependent CV curves of the (**a**) CS and (**b**) NCOS core-shell structured catalysts measured at different scan rates in non-Faradaic potential region. (**c**) "*J* vs. *v*" plots obtained at 0.05 V (*vs.* RHE) from non-Faradaic CV curves to estimate the double-layer capacitance (C_{DL}) and *ECSA*. (**d**) ECSA-corrected LSV curves for the CS and NCOS core-shell structured catalysts.

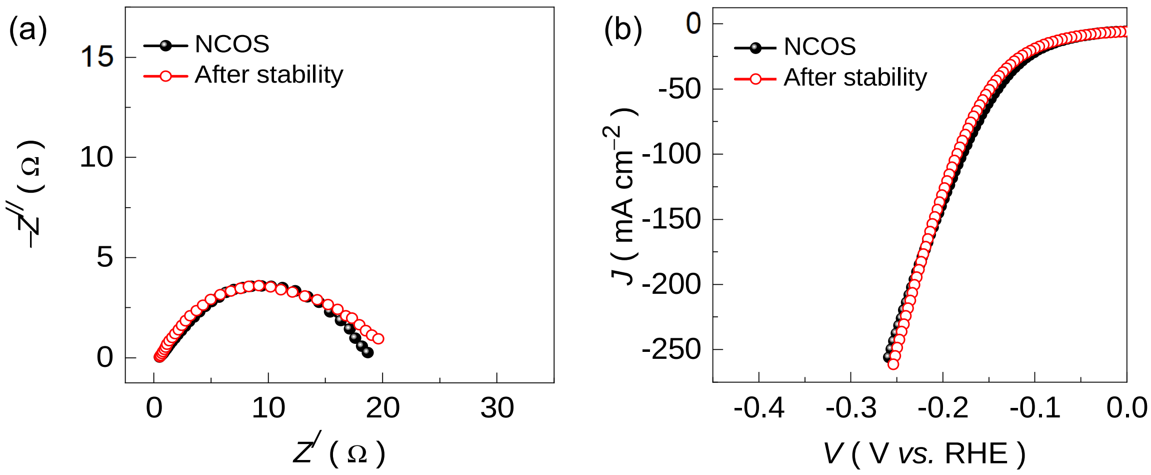

Figure S5. (**a**) EIS and (**b**) LSV curves recorded of NCOS core-shell structured catalyst recorded before and after the long-term chronopotentiometric stability.

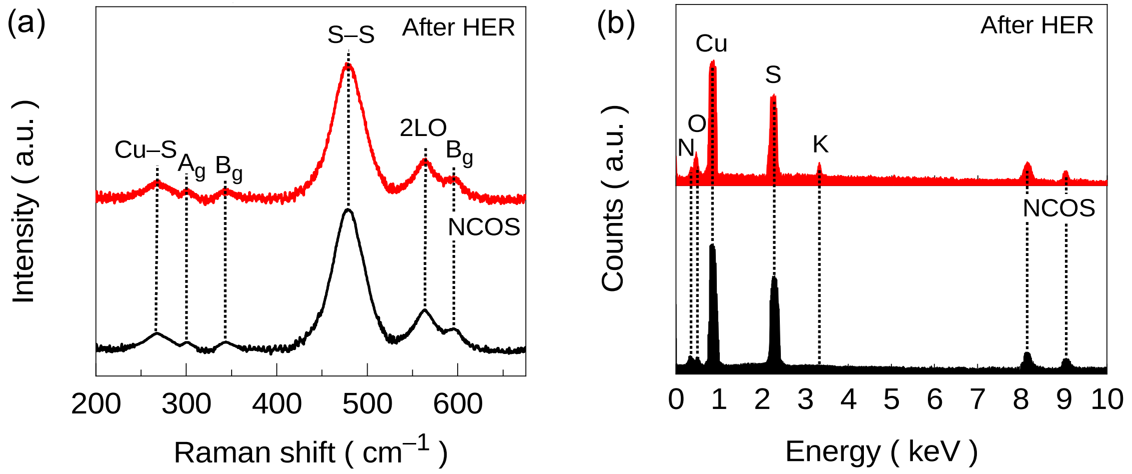

Figure S6. Post-stability measured (**a**) EDAX and (**b**) Raman spectra for the NCOS core-shell structured catalyst.
